# Prediction of Major Stroke (NIHSS > 5) Based on MR Perfusion Imaging Using a 3D ResNet

**DOI:** 10.1007/s00062-026-01623-x

**Published:** 2026-02-17

**Authors:** Christoph C. Kurmann, Adnan Mujanovic, Morin Beyeler, Leander Clénin, Philipp Becker, Roman Rohner, Richard McKinley, Shaokai Zheng, Marta Olive Gadea, Eike Immo Piechowiak, Tomas Dobrocky, Sara Pilgram-Pastor, Valerian Altersberger, Bruce Campbell, Thomas Raphael Meinel, David Seiffge, Urs Fischer, Jan Gralla, Johannes Kaesmacher

**Affiliations:** 1 Department of Diagnostic and Interventional Neuroradiology, University Hospital of Bern, Bern, Switzerland; 2 Department of Medicine and Neurology, Melbourne Brain Centre, Royal Melbourne Hospital, Melbourne, Australia; 3 Department of Neurology, University Hospital of Bern, Bern, Switzerland; 4 Support Center for Advanced Neuroimaging (SCAN), Department of Diagnostic and Interventional Neuroradiology, University Hospital of Bern, Bern, Switzerland; 5 ARTORG Center for Biomedical Engineering Research, University of Bern, Bern, Switzerland; 6 Department of Neurology, Vall d’Hebron Hospital Universitari, Barcelona, Spain; 7 Department of Neurology and Stroke Center, University Hospital of Basel, Basel, Switzerland; 8 Department of Medicine and Neurology, Melbourne Brain Centre, Royal Melbourne Hospital, Melbourne, Australia; 9 Diagnostic and Interventional Neuroradiology, Centre Hospitalier Universitaire de Tours, Tours, France

**Keywords:** Acute Ischemic Stroke, Deep Learning, MR Perfusion Imaging, National Institues of Health Stroke Scale

## Abstract

**Purpose:**

Prediction of stroke symptoms from perfusion patterns in ischemic stroke imaging would be desirable for decision making in relevant clinical scenarios as for example after incomplete mechanical thrombectomy or in perioperative strokes. Aim was to train a model to detect a major stroke defined as NIHSS > 5.

**Methods:**

Retrospective study of patients from the prospectively collected local Stroke Registry with large-/medium-vessel anterior circulation or without occlusion from 01/2015–12/2021 (N = 982, median age 74.5 (IQR 64.3–82), 47.5% female). Splits into training-set (80%), validation-set (10%) and temporally separated internal test-set (10%). Admission MR-perfusion Tmax-maps from 6 different MR-scanners were used as input for an 18-layer 3D-ResNet to predict major stroke (NIHSS > 5). Association of reported and predicted NIHSS > 5 with 90-days modified Rankin Scale (mRS) > 2 was tested using multivariate logistic regression.

**Results:**

Vessel occlusion was observed in 80% and NIHSS > 5 in 50% of all patients. On the internal test-set, Receiver Operating Characteristic area under the curve (ROC-AUC) was 0.87 (95%-CI ± 0.04) and Precision-Recall-AUC (PR-AUC) 0.85 (95%-CI ± 0.05). Accuracy was 78%, weighted F1-score 0.78. Including only vessel occlusions in the internal test-set, ROC-AUC was 0.81 (95%-CI ± 0.05) and PR-AUC 0.87 (95%-CI ± 0.05), accuracy was 73%, weighted F1-score 0.73. Reported NIHSS > 5 (aOR 2.03, 95%-CI 0.94–4.38, *p* = 0.07) and predicted NIHSS > 5 (aOR 3.61, 95%-CI 0.88–14.88, *p* = 0.08) both predicted 90-days mRS > 2, with similar model performance (BIC: 310.6 vs. 310.7; AIC: 252.4 vs. 252.5).

**Conclusion:**

Prediction of a major stroke (NIHSS > 5) from MR-perfusion Tmax-maps was feasible and performed well on our internal test set.

**Supplementary Information:**

The online version of this article (https://doi.org/10.1007/s00062-026-01623-x) contains supplementary material, which is available to authorized users.

## Introduction

The clinical severity of acute ischemic stroke is evaluated using the National Institutes of Health Stroke Scale (NIHSS), which ranges from 0 to 42 points. A NIHSS score of 0–5 is considered indicative of a minor stroke and may influence therapeutic decisions [1, 2]. The reduced blood flow in ischemic stroke to affected brain areas (hypoperfusion) can be depicted using perfusion imaging. The association of hypoperfusion of specific regional, “eloquent” brain areas and typical clinical presentation is well-known [3–5].

Based on this assumption, we hypothesize that information about location paired with the magnitude of hypoperfusion, as it is coded on MR perfusion maps, hold enough information for a deep learning model to conclude the clinically relevant impairment of acute ischemic stroke patients. The use of Artificial Intelligence plays an increasingly important role in clinical decision support and an automated objective assessment of the NIHSS could help to standardize clinical evaluation of patients, help in decision making and may ultimately improve overall patient outcomes. This would be particularly valuable in cases where patients are clinically not assessable (e.g. perioperative when intubated) or when expert neurologists are absent.

In this retrospective, exploratory study, we hypothesized that a ResNet model is capable of predicting major stroke deficits defined as NIHSS > 5 in acute ischemic stroke patients based on Time to maximum (Tmax) maps derived from MR perfusion imaging.

## Methods

### Ethics Declaration

The project was part of approved projects evaluating the association of imaging parameters with functional outcome and mortality in stroke patients (Kantonale Ethikkommission Bern, Switzerland; BASEC ID 231/14 ID 2016-01905 and 2020-01696).

### Study Design & Data

Retrospective study, consecutive patients from the prospectively collected local stroke registry of the University Hospital Bern from 01/2015–12/2021 who met the following inclusion criteria:

- Patients > 18 years of age
- Large- or medium-vessel occlusion of the anterior circulation (M1/2, A1/2) or patients without vessel occlusion
- MR perfusion imaging on admission (institutional standard of care)
- Available NIHSS assessed by a neurologist at admission
- Informed consent from the patient or the next of kin

The presence and site of occlusions were retrospectively determined by a corelab of neuroradiologists (C.K., A.M., P.B., R.R.) and vascular neurologists (M.B., L.C.), each with a minimum of one year of specialized clinical experience in neuroradiological vascular imaging. Occlusions were identified on baseline MR imaging using all available sequences. Unclear cases were resolved in joint sessions.

Imaging exams were de-identified by converting all Dicom-data to Nifti-data. Basic clinical information of the patients were retrieved from the local stroke registry.

To create a balanced dataset regarding the proportion of patients with NIHSS 0–5/> 5, patients without detectable vessel occlusion were randomly sampled from the cohort without specific matching. Balanced datasets reduce the risk of model biases, overfitting and improve performance interpretability as statistical metrics can be artificially increased by class imbalances. As we anticipate a clinical application of the model particularly in patients with perfusion deficits, we performed a separate evaluation of the internal test set only considering cases with detectable vessel occlusions.

The study has been reported according to the Checklist for Artificial Intelligence in Medical Imaging (CLAIM) [6]. All study data are available from the corresponding author upon received research plan and clearance by the ethics committee.

### Data Partition

Patients meeting the inclusion criteria were randomly divided to form a training-set (80%) and a validation-set for optimization of the model (10%). An internal test-set was created by selecting the most recent 10% of patients who arrived at the hospital, based on the chronological order of admissions in the entire study population. This approach of temporal validation, where patients of the test set are sampled at a later time point, lies in the midway of internal and external validation and is preferred to a random split of the whole population [7]. A distinct analysis of the internal test-set included only patients with detectable vessel occlusions. Data partition was disjoint at the patient level.

### Imaging

All patients underwent 1.5 T/3T MR imaging and perfusion was part of the institutional stroke protocol (Supplementary Methods I). Perfusion maps including Tmax maps were generated using an in-house algorithm that consists of a perfusion sequence regularization step and a quadratic peak-finding algorithm to calculate the surrogate Tmax map. SUPER also provides segmentations of tissue at risk using an nn-Unet. Further technical details are reported in the Supplementary Methods II and a separate work (Zheng et al., 2026 [manuscript submitted]).

### NIHSS

The NIHSS [2] of patients with clinical signs of stroke was evaluated by a neurologist upon admission to the emergency department as ground truth. The questionnaire for each patient is accessible via the local department software.

### Model and Training

Linux system server was used as platform for this study with a NVIDIA GeForce RTX 3070 graphics processing unit (8 GB of RAM).

Three-dimensional ResNet model with 18 layers was used for classification (33’223’010 trainable parameters). The fundamental architecture of ResNet was extensively described elsewhere [8]. Model parameters were randomly initialized.

Imaging input consisted of Tmax maps derived from MR perfusion imaging. Tmax maps came in different sizes (max. 256 × 256 × 34). Maps were downsampled by a factor of 2 but no sampling in the z‑axis was performed. Automated cropping was done by first creating a threshold-based mask of the brain and subsequent calculation of the center-coordinates in the x‑ and y‑axis. The center-coordinate of the z‑axis was defined as the middle slice (z/2). Cropping was performed around center-coordinates and the final dimension of all images was 80 × 95 × 18. All Tmax maps were normalized using Z‑normalization before training.

Images of the training set underwent data augmentation using the “TorchIO” package (Version 0.18.91) [9]: Blurring, noise, ghosting, as well as swapping patches within an image were randomly introduced.

Adam [10] was chosen as optimization algorithm with a mini-batch size of 4 and Cross-Entropy Loss was chosen as loss function. Learning rate was set at 5e^−5^ and cyclical learning rates were used [11]. The model was trained for 10 epochs.

Output of the model was a dichotomized classification of the NIHSS (0–5 vs. > 5).

### Evaluation

Performance of the trained model on the validation and the internal test set was assessed on confusion matrices. Accuracy, precision, recall, F1-scores for NIHSS 0–5 and NIHSS > 5, as well as the respective weighted means (by proportion of each class) were calculated. Receiver Operating Characteristic (ROC) curves and Precision-Recall (PR) curves with the corresponding area under the curve (AUC) and its 95%-confidence intervals were estimated using 1000 bootstrap samples. Gradient-weighted Class Activation Mapping (Grad-CAM) saliency maps were used to enhance interpretability of model performance [12].

A logistic regression model including age, sex, medical history of stroke, medical history of intracranial hemorrhage, hypertension, diabetes mellitus, hyperlipidemia, atrial fibrillation, smoking status, admission glucose, time from onset to imaging (min), prestroke disability (mRS), side and location of occluded vessel and volume of tissue at risk (ml, derived from SUPER segmentation) to predict admission NIHSS > 5 was used as baseline model. Critically, to address the potentially non-linear relationship between volume of tissue at risk and clinical outcome, the logistic regression was modeled using Restricted Cubic Splines (RCS) with four knots [13]. To further validate performance against a tabular machine learning benchmark, a Random Forest classifier was implemented using the above mentioned predictors. The Random Forest classifier model consisted of 100 trees with a maximum depth of five to prevent overfitting [14].

Adjusted Odds Ratios (aOR) for 90-days mRS > 2 and mRS 6 (death) were calculated from two multivariate logistic regression models including patients from the validation set and the test set (with 1 reported dichotomized NIHSS > 5 and 2) predicted NIHSS > 5 as distinct predictors. Both models were adjusted for age, sex, medical history of stroke, medical history of intracranial hemorrhage, hypertension, diabetes mellitus, hyperlipidemia, atrial fibrillation, smoking status, admission glucose, time from onset to imaging (min), prestroke disability (mRS), side and location of occluded vessel, EVT, IVT and allocation to the validation set vs. test set. Bayesian Information Criterion (BIC) and Akaike Information Criterion (AIC) were calculated to compare the performance of both models.

Frequency counts are presented as percentage, while continuous and ordinally scaled variables are shown as median and interquartile range.

All models, training and statistical analyses were implemented and conducted using Python (v3.8.8) and R (v4.0.1). All source code can be found at https://github.com/ckurmann/Prediction_of_the_NIHSS_based_on_MRP_Imaging_using_a_3DResNet/tree/main.

## Results

### Population

From January 2015–December 2021, 2039 patients presented with a large-vessel or medium-vessel occlusion of the anterior circulation. MR perfusion imaging was performed in 911 patients, documented neurology-assessed NIHSS was available shortly after or before MRI in 863. Download and processing of available MR perfusion maps was successful in 788 patients. Technical failures occurred in 75 cases (8.7%) and included corrupted DICOM headers, incomplete series transfers from the local PACS, or file format errors during NIfTI conversion that precluded successful pipeline execution. Of these cases, 41/75 (55%) had NIHSS > 5, which did not differ from the overall rate of NIHSS > 5.

**Fig. 1:**
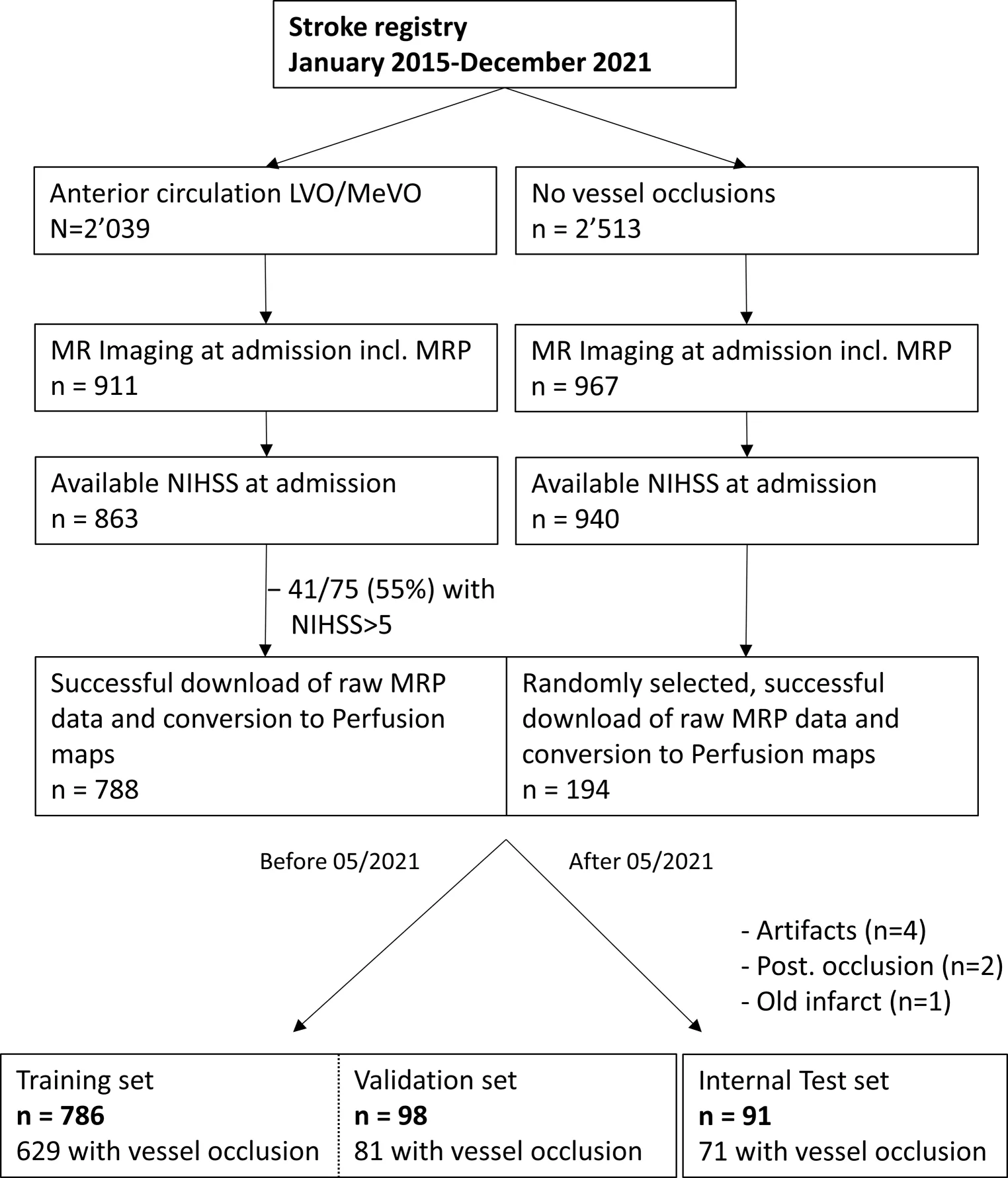
Flowchart of patient selection

To create a balanced dataset, 194 patients were randomly sampled from 940 patients with MR perfusion and available NIHSS at admission and were added to the cohort, resulting in a final cohort comprising 982 patients in total. Patients presenting before 05/2021 were randomly split into a training set (*n* = 786, 80% with vessel occlusion) and a validation set (*n* = 98, 82% with vessel occlusion). Patients presenting after 05/2021 were assigned to the internal test set (*n* = 98, 81% with vessel occlusion, Fig. 1).

After careful inspection of the internal test set before testing, seven patients were excluded: Four patients with artifacts, two patients with infarcts in the territory of the posterior cerebral artery and one patient with a large old infarct in the territory of the middle cerebral artery (5/7 with NIHSS > 5, Supplementary Figure I).

Median age was 74.7 years (IQR 64.7–83.1) in the training set, 75.8 years (IQR 83.7–81.5) in the validation set and 75.1 years (IQR 63.1–81.7) in the internal test set. NIHSS > 5 was present in 51% of the training set, 49% of the validation set and 42.9% of the internal test set (Table 1). When comparing the individual sets, statistically significant differences occurred in proportion of female participants (48.1% vs. 56.1% vs. 36.3%, *p* = 0.02) and hyperlipidemia (67.3% vs. 71.4% vs. 82.4%, *p* = 0.01).

**Table 1:** Patient characteristics

	Training set	Validation set	Internal test set	*p*
*n*	786	98	91	–
Age (median [IQR])	75 [65, 83]	76 [64, 81]	75 [63, 82]	0.795
Female (%)	378 (48.1)	55 (56.1)	33 (36.3)	0.022
NIHSS > 5 (%)	401 (51.0)	48 (49.0)	39 (42.9)	0.329
Medical history (%)				
Stroke	116 (15.3)	10 (10.9)	9 (9.9)	0.228
Transient ischemic attack	37 (4.9)	4 (4.3)	7 (7.7)	0.487
Intracranial hemorrhage	9 (1.2)	0 (0.0)	1 (1.1)	0.576
Hypertension	559 (73.7)	66 (71.7)	63 (69.2)	0.625
Diabetes	134 (17.7)	19 (20.9)	14 (15.4)	0.619
Hyperlipidemia	508 (67.3)	65 (71.4)	75 (82.4)	0.011
Smoking	151 (21.3)	16 (17.8)	19 (20.9)	0.738
Atrial Fibrillation	261 (34.5)	34 (37.4)	25 (27.5)	0.321
Admission blood glucose (mmol/L, median (SD))	6.90 (2.12)	6.73 (2.11)	7.20 (2.68)	0.312
Time from onset to imaging (min, median [IQR])	248 [118, 607]	230 [102, 567]	256 [129, 571]	0.855
Vessel Occlusion (%)	–	–	–	0.797
No occlusion	157 (20.0)	17 (17.3)	20 (22.0)	–
Intracranial ICA	13 (1.7)	0 (0.0)	1 (1.1)	–
T-/L-occlusion of ICA	55 (7.0)	5 (5.1)	3 (3.3)	–
M1-segment	251 (31.9)	32 (32.7)	31 (34.1)	–
M2-segment	307 (39.1)	43 (43.9)	36 (39.6)	–
A1-segment	3 (0.4)	1 (1.0)	0 (0.0)	–
Side of Vessel Occlusion—left (%)	314 (39.9)	40 (40.8)	28 (30.8)	0.486
Magnetic Field Strength (3 T) (%)	276 (35.1)	37 (37.8)	38 (41.8)	0.427

*IQR—*Interquartile range*, SD—*Standard deviation, *NIHSS—*National Institutes of Health Stroke Scale, *ICA—*Internal carotid artery

## Performance Metrics

**Fig. 2:**
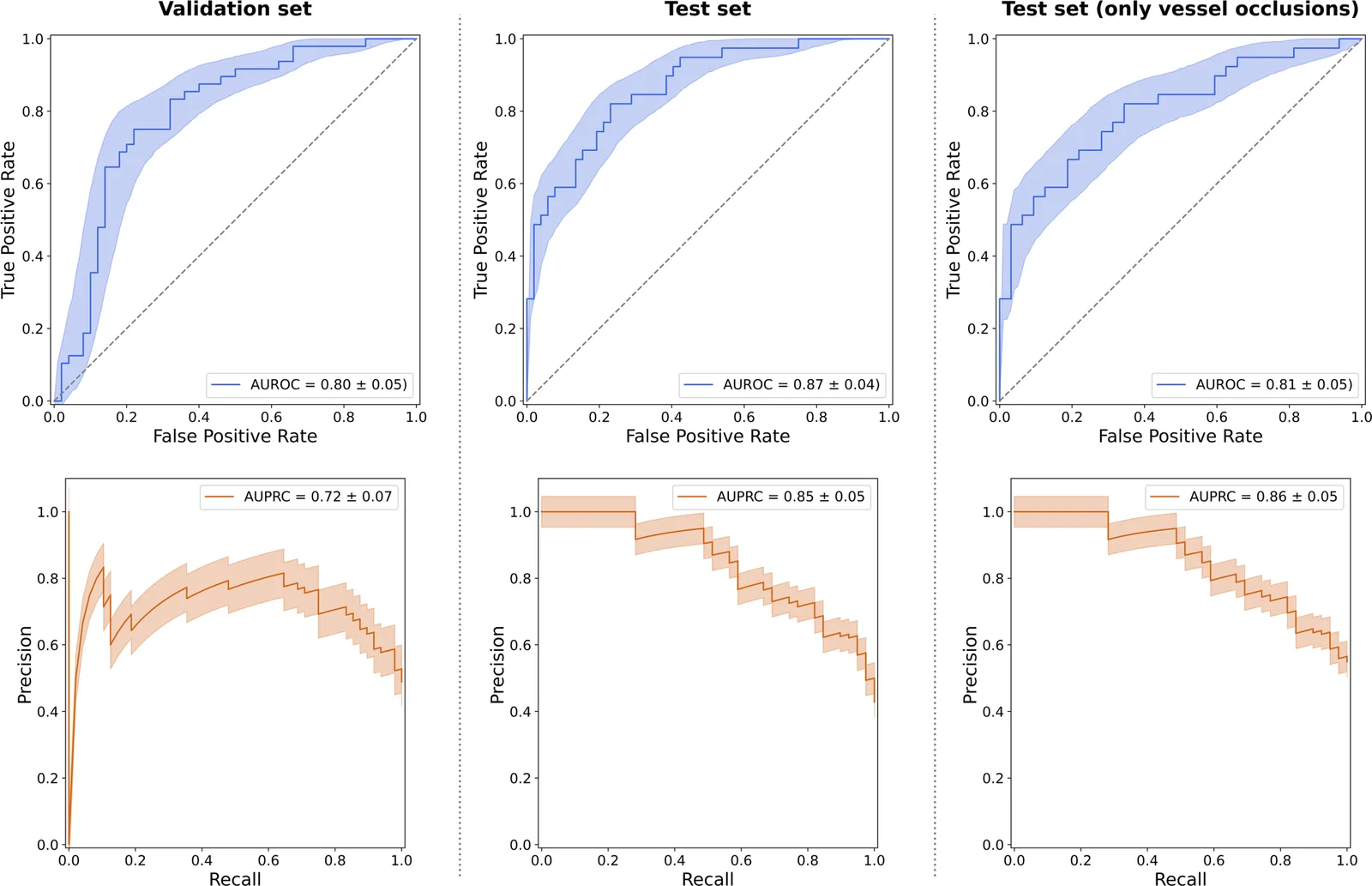
Receiver Operating Characteristic with corresponding Area under the Curve values (AUC, top) and Precision-Recall curve with corresponding AUC (bottom) for the validation set (left), the internal test set (middle) and the test set when only including vessel occlusions (right)

ROC-AUC (validation set 0.80 ± 0.05, internal test set 0.87 ± 0.04) and PR-AUC (validation set 0.72 ± 0.07, internal test set 0.85 ± 0.05) were higher for the internal test set (Fig. 2). Other performance metrics of the model were also slightly better for the internal test set than for the validation set (accuracy 78% vs. 76%, weighted precision 0.78 vs. 0.76, weighted recall 0.78 vs. 0.76 and weighted F1-score 0.78 vs. 0.85; Table 2).

**Table 2:** Performance metrics of the validation set, the internal test set and the internal test set only including cases with vessel occlusions

	Validation set	Internal test set	Internal test set (only vessel occlusions)
Accuracy	0.76	0.78	0.73
Precision			
NIHSS 0–5	0.81	0.82	0.71
NIHSS > 5	0.71	0.73	0.75
*Weighted average*	*0.76*	*0.78*	*0.73*
Recall			
NIHSS 0–5	0.68	0.79	0.69
NIHSS > 5	0.83	0.77	0.77
*Weighted average*	*0.76*	*0.78*	*0.73*
F1-score			
NIHSS 0–5	0.74	0.80	0.70
NIHSS > 5	0.77	0.75	0.76
*Weighted average*	*0.75*	*0.78*	*0.73*
ROC-AUC (95%-CI)	0.80 (± 0.05)	0.87 (± 0.04)	0.81 (± 0.05)
PR-AUC (95%-CI)	0.72 (± 0.07)	0.85 (± 0.05)	0.86 (± 0.05)

*NIHSS*—National Institutes of Health Stroke Scale, *ROC-AUC*—Receiver Operating Characteristic area under the curve, *PR-AUC*—Precision-Recall area under the curve, *CI*—Confidence intervals

When only including patients with detectable vessel occlusions of the internal test set, ROC-AUC was 0.81 ± 0.05 and PR-AUC 0.86 ± 0.05 (Fig. 2). Accuracy was 73%, weighted precision 0.73, weighted recall 0.73, weighted F1-score 0.73. Confusion matrices for each group can be found in Supplementary Figures II and III.

Subgroup analyses demonstrated consistent model performance across different magnetic field strengths (1.5 T vs. 3 T, see Supplementary Tables 1/2).

The 3D ResNet demonstrated superior discrimination compared to both the multivariable logistic regression using Restricted Cubic Splines (LR RCS) and the Random Forest classifier. On the internal test set, the LR RCS model achieved an ROC-AUC of 0.67 ± 0.13, in the subgroup including only vessel occlusions, ROC-AUC dropped to 0.50 ± 0.13. The Random Forest classifier showed improved performance over the regression baseline, with an internal test set ROC-AUC of 0.74 ± 0.09 and for the vessel occlusion subgroup a ROC-AUC of 0.57 ± 0.13 (Supplementary Table 3).

## Saliency Maps and Analysis of Misclassifications

**Fig. 3:**
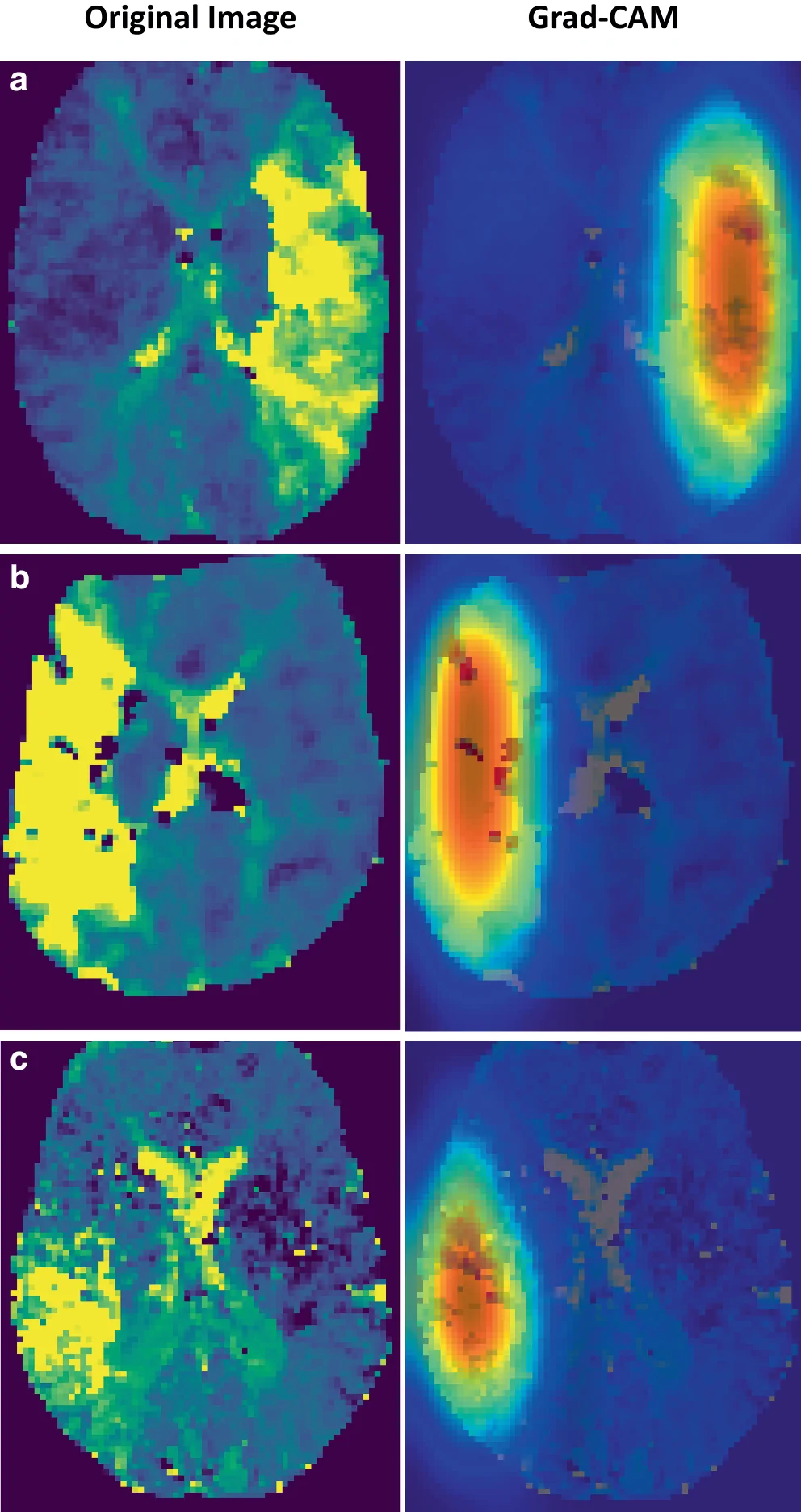
Examples of correctly classified cases of the internal test set. Left: Original input images (Tmax maps) with hypoperfused areas, Right: Overlayed saliency maps generated with Grad-CAM from the 3D ResNet-18 model. **a** M1-occlusion left, **b** M1-occlusion right, **c** M2-occlusions right

Saliency maps derived from the trained 3D ResNet-18 model were generated using Grad-CAM to visualize the regions of highest importance for the model’s decision-making process. These maps employ a color-coded scheme where red indicates areas of maximum relevance to the model’s output, enhancing the interpretability and explainability of the neural network’s decision-making process. Saliency maps often identified hypoperfused areas in correct classifications (Fig. 3).

**Fig. 4:**
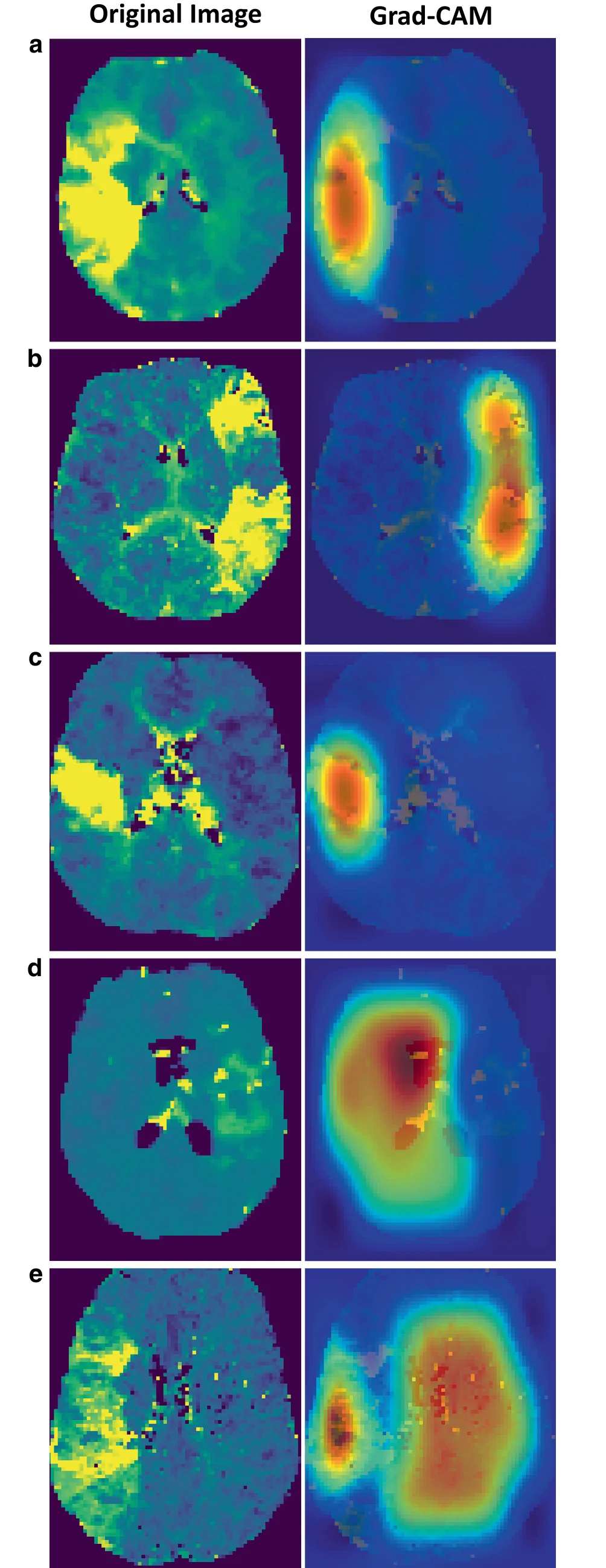
Misclassified cases of the internal test set. The original input Tmax map derived from MR perfusion imaging is shown for each case on the left side while the corresponding saliency maps generated with Grad-CAM from the 3D ResNet-18 model are displayed on the right side. **a** M1-occlusion right (NIHSS 4), **b** M2-occlusion left with subacute infarct in anterior middle cerebral artery territory (NIHSS 4), **c** M2-occlusion right (NIHSS 6), **d** M2-occlusion left (NIHSS 8), **e** M1-occlusion right (NIHSS 4)

Figure 4 shows exemplary saliency maps of misclassified cases from the internal test set. Figure 4a, b show cases with relatively large perfusion deficits but NIHSS 4. On the other hand, Fig. 4c shows a smaller perfusion deficit with a NIHSS 6, showing the importance of subtle differences of location and size of hypoperfusion for the resulting clinical deficit. However, saliency maps were completely off of hypoperfused brain areas in some other cases (Fig. 4d, e), maybe due to confounders that are not readily discernible through visual inspection.

Of the 20 misclassified cases of the internal test set, 9 had a NIHSS of 5 ± 1, being close to the threshold. Of the remaining cases, all three misclassified patient that presented with NIHSS of < 3 had a M1-occlusion.

## Association with Functional Outcome

For the patients of the validation set and the internal test set, the aOR for a 90-day mRS > 2 was 2.43 (95% CI: 1.00–5.87, *p* = 0.05) for patients with a reported NIHSS > 5, and 1.94 (95% CI: 0.85–4.42, *p* = 0.12) for those with a predicted NIHSS > 5 (detailed results see Supplementary Tables 4/5). Comparing both models, there was no relevant difference between the BIC values (with reported NIHSS > 5: 275.7 vs. with predicted NIHSS > 5: 277.5) and the AIC values (with reported NIHSS > 5: 219.7 vs. with predicted NIHSS > 5: 221.5).

The aOR for death at 90 days was 1.10 (95% CI: 0.30–4.30, *p* = 0.89) for patients with a reported NIHSS > 5, and 1.92 (95% CI: 0.50–7.38, *p* = 0.34) for patients with predicted NIHSS > 5 (BIC 187.2 vs. 186.3, AIC 131.2 vs. 130.3, see Supplementary Tables 6/7).

## Discussion

Our study shows that dichotomized prediction of NIHSS > 5 of patients with suspected acute ischemic stroke based on Tmax maps using a 3D ResNet was feasible in our cohort.

While works by Pinto et al. [15] and from the ISLES challenge [16] have successfully utilized convolutional neural networks (CNNs) to predict final infarct volume and voxel-wise lesion expansion, these models primarily address the radiological evolution of the stroke. Our study differentiates itself from the prevailing literature by shifting the predictive target from radiological ‘tissue fate’ to direct clinical presentation. Second, we contrast our methodology with traditional Lesion-Symptom Mapping (LSM). Recent studies, such as those by Garrard et al. [3] and Ding et al. [4], provide vital insights into eloquent brain regions using voxel-based statistical correlations. However, our implementation utilizes a 3D-ResNet architecture to perform deep spatial feature extraction, moving beyond the linear or voxel-wise assumptions of traditional LSM.

The results from our 3D CNN-based model with Tmax maps derived from MR perfusion and explicitly predicting the NIHSS primarily serve as proof-of-concept, suggesting the potential of the model for improved performance with larger training cohorts and its applicability to similar tasks.

We used a relatively simple and small deep learning architecture and trained it from random initialization. We hypothesize that only a considerably larger sample size would profit from more complex architectures and more sophisticated training methods. Compared to the validation set and the whole internal test set, the model performed slightly worse when only including patients with vessel occlusions of the internal test set. This indicates that the model easily recognized and classified cases without perfusion deficits. The model had difficulty in patients with M1-occlusions, who have large perfusion deficits, but presented with low NIHSS scores. Since M1-occlusions are generally linked to higher NIHSS scores, the model’s ability to detect the less common cases with low NIHSS scores could improve with more training data. These cases also highlight how small differences in hypoperfusion patterns can affect clinical symptoms.

The performance disparity observed between our 3D ResNet and the baseline spline-based logistic regression model and Random Forest classifier suggests that the 3D deep-learning architecture successfully extracts complex, spatial relationships. The performance gap indicates that the model identifies functional brain eloquence as a potent predictor of clinical status instead of mere vessel territory or aggregate tissue at risk volume. This interpretation is further corroborated by Grad-CAM saliency maps, which visually demonstrate that the model’s ‘attention’ prioritizes specific regional hypoperfused brain regions when making predictions, a finding that aligns with clinical intuition and supports the model’s interpretability. Visual representations of saliency maps generated with Grad-CAM offer potential utility in clinical settings, enabling physicians to understand the basis of the model’s predictions. Such interpretability may enhance more informed decision-making regarding the reliability and applicability of the model’s output in individual cases, bridging the gap between model predictions and clinical judgment.

In analyses of unfavorable functional outcome, defined as a 90-day mRS > 2 and mRS 6, the adjusted regression model incorporating predicted NIHSS > 5 performed comparably to the model using reported NIHSS > 5. This serves as indirect validation of the model, also showing that the predictions correlated with 90-days mRS > 2, although being non-significant. Larger cohorts are needed to confirm these exploratory results.

Furthermore, it needs to be mentioned that the surrogate Tmax maps utilized in this study were generated via a deconvolution-free algorithm, making them conceptually similar to relative Time-to-Peak (rTTP) maps, which have been shown to provide lesion volumes comparable to standard Tmax while remaining robust against the variability and noise inherent in Arterial Input Function (AIF) selection [17].

## Applications in Clinical Settings

We propose a use case where neuro-/radiologists send perfusion maps showing a perfusion deficit to our model, enabling NIHSS score estimation to support informed therapeutic decision-making. Automated assessment of the NIHSS could standardize neurological patient evaluation with acute ischemic stroke and assist the treating physicians, especially in situations where patients are not adequately clinically assessable. A prominent example would be intubated patients and notably, the risk of perioperative strokes can rise up to 5–10% in high-risk cardiac or major vascular surgeries [18]. It also could be useful in absence of NIHSS-certified neurologists in the light of general shortage of neurologists [19].

Although CT perfusion is generally more widely utilized in acute neuroimaging, this study employed MR perfusion in accordance with our institutional diagnostic protocol. At our center, MR imaging including perfusion imaging serves as the first-line modality for the evaluation of suspected ischemic stroke. Using only MR perfusion ensured comprehensive evaluation within a specialized stroke-center workflow. However, our model has to be adapted for use with other imaging techniques, which we hypothesize will be possible through retraining and transfer learning. This could be particularly useful in cases of incomplete mechanical thrombectomy. In such scenarios, interventionalists could perform flat-panel CT perfusion imaging immediately after the procedure directly in the angiography suite [20–22]. The resulting perfusion maps could then be used to predict the likely neurological deficit. Objective information about the importance of remaining hypoperfused brain areas would be extremely valuable in deciding whether to continue thrombectomy or administer additional intra-arterial thrombolysis [23], or if the risks of further interventions outweigh the potential benefits. Currently, our algorithm has been trained and validated exclusively on MR-derived Tmax maps and several technical challenges must be addressed before implementation of this model in the angiography suite. Furthermore, it is important to mention that flat-panel CT perfusion imaging is a relatively new application and most existing evidence is derived from pilot studies and practical challenges and potential benefits need to be addressed in future studies [24, 25].

We also think that a similar model could be used to not only predict the evolution of hypoperfused brain tissue, as it has been done in several other studies [15, 26], but also to predict clinical outcome of the patients and help in prognostication. The need for such assessment has become even more apparent following recent results of the randomized controlled trials ESCAPE-MeVO [27], DISTAL [28] and DISCOUNT [29] which could not show clinical benefit of mechanical thrombectomy in medium and distal vessel occlusions. These results highlight the importance of improving patient selection in such cases, where assessing the functional significance of hypoperfused brain areas could be essential for better treatment decisions and ultimately better patient outcomes.

Our primary goal for future research is the adaptation of this model for flat-panel CT perfusion imaging, facilitating real-time clinical decision support during EVT. In order to achieve this end goal, future projects should incorporate CT perfusion/flat-panel detector CT perfusion images, other perfusion maps, distal occlusions/occlusions of the posterior circulation as well as patients from different centers. Another important step for future models should be the ability to predict continuous NIHSS scores. These advancements would support more personalized and effective treatment strategies for patients with wide spread applicability.

## Limitations

Several limitations regarding the generalizability of our findings must be acknowledged. The model was tested using temporal validation, which lies in the midway of internal and external validation and is a reasonable alternative when lacking data from another center [7]. However, external validation is essential to assess a predictive model and warrants future studies. Although included consecutively, the potential for selection bias must be considered, as we used a highly selected, retrospective and monocentric patient population. NIHSS was missing for a considerable portion of patients, for example in the most severe cases probably due to intubation on admission, which may limit the model’s representation of the full clinical spectrum of acute stroke.

Regarding methodological constraints, the scope of this proof-of-concept was limited to large- and medium-vessel occlusions of the anterior circulation. The isolated use of Tmax maps as the primary imaging input, while optimized for model training, may have resulted in the loss of potentially useful physiological information contained within other perfusion parameters such as cerebral blood flow (CBF) or cerebral blood volume (CBV). We omitted diffusion-weighted imaging (DWI) data from the predictive model. While this allowed for an isolated assessment of the perfusion signature’s independent prognostic value, it may precluded an analysis of the synergistic effect between established infarct core volume and spatial hypoperfusion on clinical outcomes. Finally, the exclusion of scans with significant artifacts and large chronic infarcts likely improved performance metrics, though this remains an inherent limitation of the imaging modality that clinicians must consider when interpreting model outputs.

Finally, the use of the NIHSS as a ground truth introduces specific challenges. The current model performs a relatively simple dichotomized prediction (NIHSS > 5), which overlooks the nuanced clinical significance of specific neurological deficits. While we have categorized a score of > 5 as a “major stroke”, we acknowledge that lower scores can still correspond to clinically significant and disabling deficits—such as isolated aphasia or neglect—which are not explicitly captured by our current framework. Furthermore, the binary threshold treats a misclassification near the cutoff (e.g., ± 1 point) with the same weight as a more substantial discrepancy, despite the inherent difficulty of classification in this narrow margin. Lastly, the reliance on the NIHSS is subject to considerable interrater variation among neurologists [30], which may introduce inherent noise into the predictive training process.

## Conclusion

This proof-of-concept study found that binary classification of NIHSS based on MR Tmax perfusion maps was feasible and performed well on our internal test set. Larger cohorts for training of the model, external testing and examination of its general utility for supporting decision-making in absence of neurologists or when clinical examination is not possible are warranted. Application on other imaging modalities and further investigation into prediction of clinical outcomes seem a promising approach.

## Supplementary Information

ESM1 Supplemental Material 1

ESM2 Supplemental Material 2

## Data Availability

All study data are available from the corresponding author upon received research plan and clearance by the ethics committee.
