# Supplementary material for: Prediction of Major Stroke (NIHSS > 5) Based on MR Perfusion Imaging Using a 3D ResNet
Source: Clin Neuroradiol. 2026 Feb 17;36(2):661–71. doi: 10.1007/s00062-026-01623-x (PMC13687120; doi:10.1007/s00062-026-01623-x)
Supplement: Supplementary file 1 [file 62_2026_1623_MOESM1_ESM.pdf]

# Checklist for Artificial Intelligence in Medical Imaging (CLAIM): 2024 Update

| Section / Topic | No. | Item | Page / Line | No | NA |
|---|---|---|---|---|---|
| **TITLE / ABSTRACT** | | | | | |
| | 1 | Identification as a study of AI methodology, specifying the category of technology used (e.g., deep learning) | 1 | | |
| **ABSTRACT** | | | | | |
| | 2 | Summary of study design, methods, results, and conclusions | 2 | | |
| **INTRODUCTION** | | | | | |
| | 3 | Scientific and/or clinical background, including the intended use and role of the AI approach | 4 | | |
| | 4 | Study aims, objectives, and hypotheses | 4 | | |
| **METHODS** | | | | | |
| ***Study Design*** | 5 | Prospective or retrospective study | 4 | | |
| | 6 | Study goal | 4 | | |
| ***Data*** | 7 | Data sources | 4 | | |
| | 8 | Inclusion and exclusion criteria | 4 | | |
| | 9 | Data pre-processing | 4 | | |
| | 10 | Selection of data subsets | 4 | | |
| | 11 | De-identification methods | 4 | | |
| | 12 | How missing data were handled | 4 | | |
| | 13 | Image acquisition protocol | 5 | | |
| ***Reference Standard*** | 14 | Definition of method(s) used to obtain reference standard | 5 | | |
| | 15 | Rationale for choosing the reference standard | - | | X |
| | 16 | Source of reference standard annotations | 5 | | |
| | 17 | Annotation of test set | 5 | | |
| | 18 | Measures of inter- and intra-rater variability of features described by the annotators | - | | X |
| ***Data Partitions*** | 19 | How data were assigned to partitions | 5 | | |
| | 20 | Level at which partitions are disjoint | 5 | | |
| ***Testing Data*** | 21 | Intended sample size | 4 | | |

| Section / Topic | No. | Item | Page / Line | No | NA |
|---|---|---|---|---|---|
| *Model* | **22** | Detailed description of model | **5** | | |
| | **23** | Software libraries, frameworks, and packages | **5** | | |
| | **24** | Initialization of model parameters | **5** | | |
| *Training* | **25** | Details of training approach | **5/6** | | |
| | **26** | Method of selecting the final model | **6** | | |
| | **27** | Ensembling techniques | **-** | | **X** |
| *Evaluation* | **28** | Metrics of model performance | **6** | | |
| | **29** | Statistical measures of significance and uncertainty | **-** | | **X** |
| | **30** | Robustness or sensitivity analysis | **-** | | **X** |
| | **31** | Methods for explainability or interpretability | **6** | | |
| | **32** | Evaluation on internal data | **5** | | |
| | **33** | Testing on external data | **-** | | **X** |
| | **34** | Clinical trial registration | **-** | | **X** |
| **RESULTS** | | | | | |
| *Data* | **35** | Numbers of patients or examinations included and excluded | **7** | | |
| | **36** | Demographic and clinical characteristics of cases in each partition | **7, Tab1** | | |
| *Model performance* | **37** | Performance metrics and measures of statistical uncertainty | **7, Tab2** | | |
| | **38** | Estimates of diagnostic performance and their precision | **-** | | **X** |
| | **39** | Failure analysis of incorrect results | **7/8, Suppl.** | | |
| **DISCUSSION** | | | | | |
| | **40** | Study limitations | **9/10** | | |
| | **41** | Implications for practice, including intended use and/or clinical role | **7-9** | | |
| **OTHER INFORMATION** | | | | | |
| | **42** | Provide a reference to the full study protocol or to additional technical details | **6** | | |
| | **43** | Statement about the availability of software, trained model, and/or data | **6** | | |
| | **44** | Sources of funding and other support; role of funders | **11** | | |

* Indicate page and/or line number for each checklist item that is present.  NA = not applicable.

Tejani AS, Klontzas ME, Gatti AA, et al.  Checklist for Artificial Intelligence in Medical Imaging (CLAIM):2024 Update. Radiol Artif Intell 2024;6(4):e240300. https://doi.org/10.1148/ryai.240300
