# Supplementary material for: Prediction of Major Stroke (NIHSS > 5) Based on MR Perfusion Imaging Using a 3D ResNet
Source: Clin Neuroradiol. 2026 Feb 17;36(2):661–71. doi: 10.1007/s00062-026-01623-x (PMC13687120; doi:10.1007/s00062-026-01623-x)
Supplement: Supplementary file 2 [file 62_2026_1623_MOESM2_ESM.pdf]

Prediction of disabling stroke (NIHSS >5) based on MR Perfusion Imaging using a 3D ResNet

-

Supplemental Material

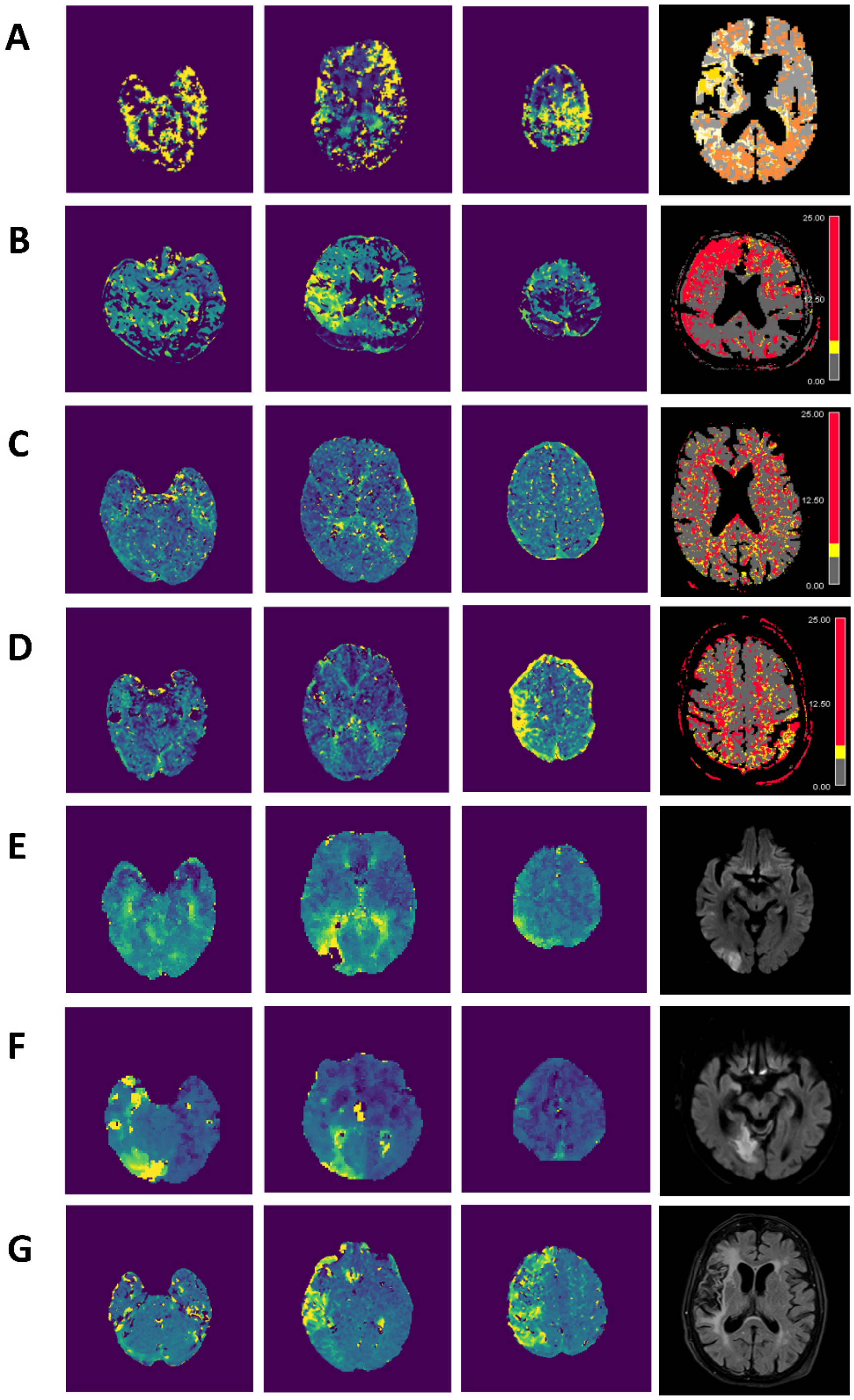

Supplementary Figure I Excluded cases from the internal test set. A-D: Artifacts, with corresponding Tmax perfusion maps processed with OLEA software as comparison. E/F: Infarcts in territory of posterior cerebral artery (PCA) with corresponding DWI images. G: Old infarct in middle cerebral artery territory right with corresponding FLAIR image.

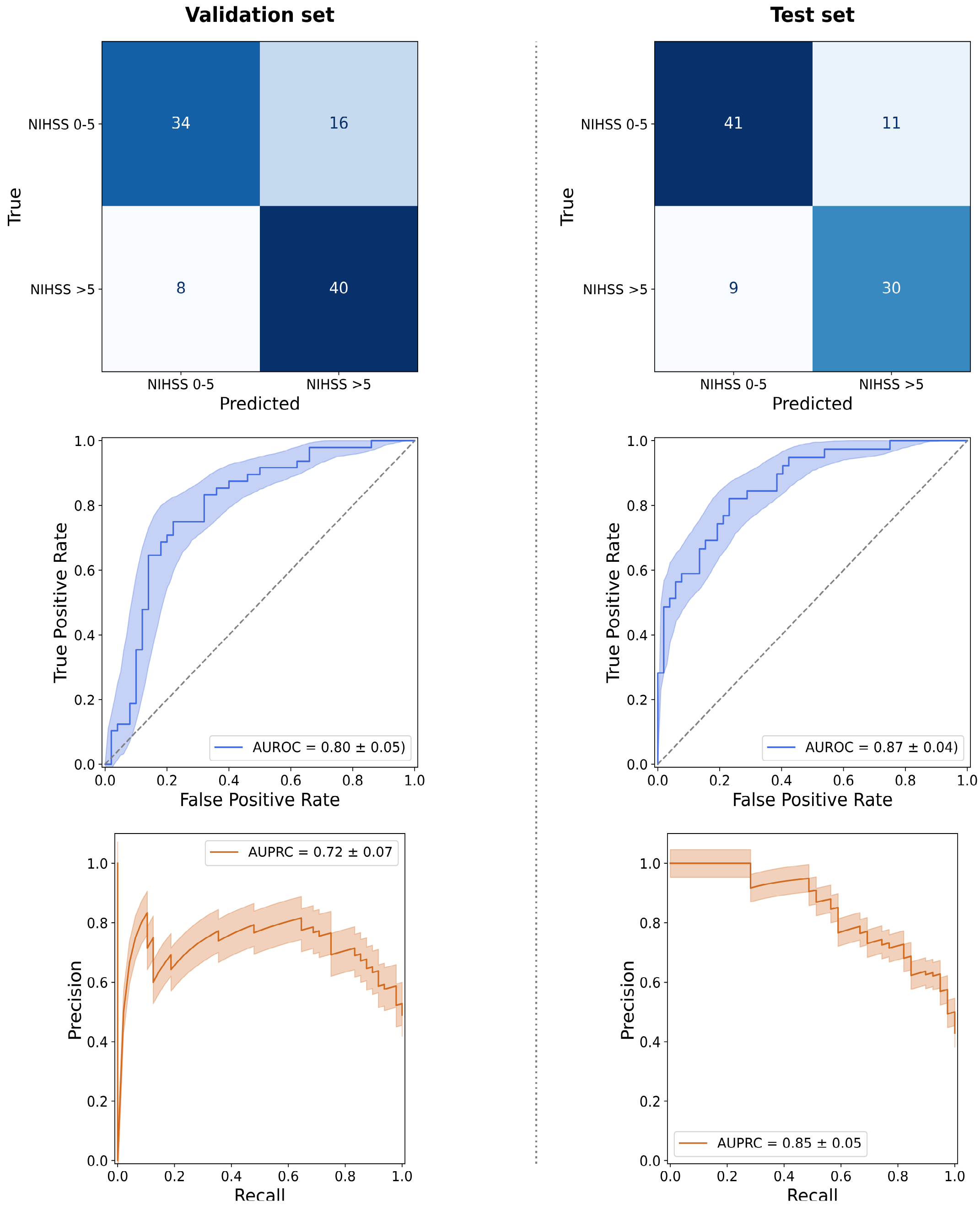

Supplementary Figure II Left: Confusion matrix (top), Receiver Operating Characteristic with corresponding Area under the Curve values (AUC, middle) and Precision-Recall curve with corresponding AUC (bottom) for the validation set; Right: Confusion matrix (top), Receiver Operating Characteristic with corresponding AUC values (middle) and Precision-Recall curve with corresponding AUC (bottom) for the internal test set.

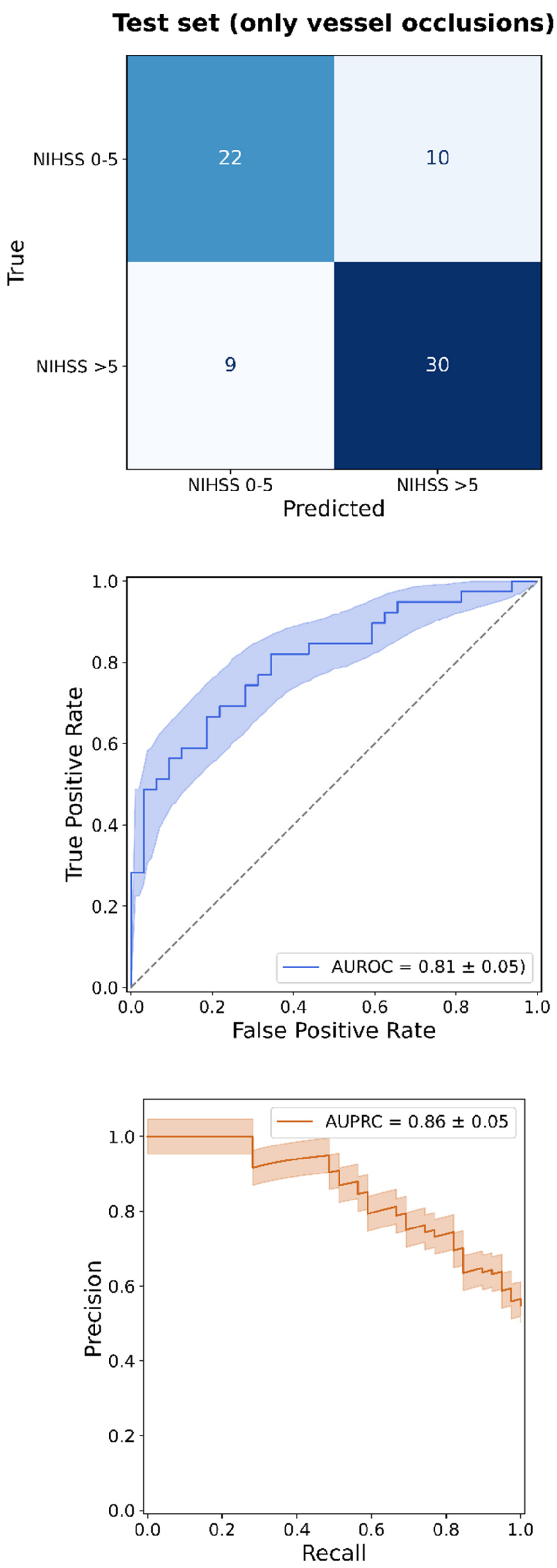

Supplementary Figure III Confusion matrix (top), Receiver Operating Characteristic with corresponding Area under the Curve values (AUC, middle) and Precision-Recall curve with corresponding AUC (bottom) for the internal test set only including cases with vessel occlusion.

Supplementary Table I Model performance metrics for the internal test set stratified by magnetic field strength (3T)

	**Internal test set**	**Internal test set (only vessel occlusions)**
Accuracy	0.72	0.69
Precision	0.71	0.75
Recall	0.67	0.67
F1-score	0.69	0.71
ROC-AUC (95%-CI)	0.87 (± 0.09)	0.83 (± 0.12)
PR-AUC (95%-CI)	0.88 (± 0.09)	0.89 (± 0.09)

Supplementary Table II Model performance metrics for the internal test set stratified by magnetic field strength (1.5T)

	**Internal test set**	**Internal test set (only vessel occlusions)**
Accuracy	0.84	0.79
Precision	0.75	0.75
Recall	0.90	0.90
F1-score	0.81	0.81
ROC-AUC (95%-CI)	0.89 (± 0.09)	0.83 (± 0.13)
PR-AUC (95%-CI)	0.85 (± 0.11)	0.85 (± 0.11)

Supplementary Table III Results of the logistic regression model using Restricted Cubic Splines (LR RCS) and Random Forest classifier (RF) to predict NIHSS >5 in the combined validation and test set, in the test set alone and in the test set only including vessel occlusions

	**Validation + Internal test set**	**Internal test set**	**Internal test set (only vessel occlusions)**
**LR RCS**			
Accuracy	0.59	0.55	0.42
Precision (NIHSS >5)	0.55	0.47	0.47
Recall (NIHSS >5)	0.59	0.44	0.44
F1-score (NIHSS >5)	0.57	0.45	0.45
ROC-AUC (95%-CI)	0.68 (± 0.07)	0.67 (± 0.13)	0.50 (± 0.13)
PR-AUC (95%-CI)	0.59 (± 0.09)	0.58 (± 0.14)	0.59 (± 0.15)
**RF**			
Accuracy	0.71	0.68	0.59
Precision (NIHSS >5)	0.64	0.60	0.60
Recall (NIHSS >5)	0.83	0.74	0.74
F1-score (NIHSS >5)	0.72	0.67	0.67
ROC-AUC (95%-CI)	0.78 (± 0.08)	0.74 (± 0.09)	0.57 (± 0.13)
PR-AUC (95%-CI)	0.72 (± 0.11)	0.65 (± 0.15)	0.65 (± 0.15)

Supplementary Table IV Adjusted Odds Ratios for 90-days mRS >2 of multivariate logistic regression including reported NIHSS >5

	aOR	95% CI lower	95% CI upper	p
**Actual NIHSS >5**	**2.43**	**1.00**	**5.87**	**0.05**
Age	1.03	0.99	1.07	0.12
Female sex	1.22	0.55	2.69	0.63
MedHist stroke	2.18	0.59	8.03	0.24
MedHist AHT	2.22	0.89	5.60	0.09
Diabetes	0.62	0.21	1.76	0.37
Smoking status	0.60	0.22	1.68	0.33
Hyperlipidemia	0.68	0.27	1.72	0.42
Atrial fibrillation	0.58	0.25	1.39	0.22
First Glucose (mmol/l)	1.01	0.85	1.20	0.90
Onset to imaging (min)	1.00	0.99	1.00	0.17
Prestroke mRS	1.35	0.88	2.06	0.17
Side of vessel occlusion	1.20	0.57	2.50	0.63
Affected vessel	1.33	0.91	1.93	0.14
EVT	1.50	0.45	4.99	0.51
IVT	0.75	0.34	1.67	0.49
Validation/test set	0.79	0.36	1.72	0.55

aOR adjusted Odds Ratios; *CI* Confidence Intervals; *NIHSS* National Institutes of Health Stroke Scale; *ICH* Intracerebral hemorrhage; *AHT* Arterial hypertension; *ICH* Intracranial hemorrhage; *mRS* modified Ranking Scale; *EVT* Endovascular thrombectomy; *IVT* Intravenous thrombolysis

Supplementary Table V Adjusted Odds Ratios for 90-days mRS >2 of multivariate logistic regression including predicted NIHSS >5

	OR	95% CI lower	95% CI upper	p
**Predicted NIHSS >5**	**1.94**	**0.85**	**4.42**	**0.12**
Age	1.03	0.99	1.07	0.09
Female sex	1.16	0.53	2.55	0.70
MedHist stroke	2.60	0.73	9.31	0.14
MedHist AHT	2.13	0.85	5.30	0.11
Diabetes	0.63	0.22	1.82	0.39
Smoking status	0.62	0.23	1.71	0.36
Hyperlipidemia	0.64	0.26	1.60	0.35
Atrial fibrillation	0.69	0.30	1.60	0.39
First Glucose (mmol/l)	1.00	0.85	1.20	0.90
Onset to imaging (min)	1.00	0.99	1.00	0.14
Prestroke mRS	1.31	0.86	2.00	0.21
Side of vessel occlusion	1.29	0.62	2.72	0.49
Affected vessel	1.34	0.92	1.95	0.13
EVT	1.99	0.63	6.19	0.24
IVT	0.80	0.37	1.78	0.59
Validation/test set	0.79	0.37	1.73	0.56

aOR adjusted Odds Ratios; *CI* Confidence Intervals; *NIHSS* National Institutes of Health Stroke Scale; *ICH* Intracerebral hemorrhage; *AHT* Arterial hypertension; *ICH* Intracranial hemorrhage; *mRS* modified Ranking Scale; *EVT* Endovascular thrombectomy; *IVT* Intravenous thrombolysis

Supplementary Table VI Adjusted Odds Ratios for death at 90 days (mRS 6) of multivariate logistic regression including reported NIHSS >5

	aOR	95% CI lower	95% CI upper	p
**Actual NIHSS >5**	**1.10**	**0.30**	**4.30**	**0.89**
Age	1.07	1.00	1.14	0.04
Female sex	0.96	0.28	3.34	0.95
MedHist stroke	0.24	0.02	2.70	0.25
MedHist AHT	1.84	0.40	8.50	0.44
Diabetes	0.97	0.21	4.49	0.97
Smoking status	1.04	0.21	5.17	0.96
Hyperlipidemia	0.56	0.15	2.07	0.38
Atrial fibrillation	1.41	0.42	4.74	0.58
First Glucose (mmol/l)	1.08	0.87	1.34	0.48
Onset to imaging (min)	1.00	0.99	1.00	0.10
Prestroke mRS	1.61	0.94	2.75	0.08
Side of vessel occlusion	2.23	0.70	7.08	0.17
Affected vessel	0.96	0.50	1.83	0.90
EVT	5.84	0.50	68.39	0.16
IVT	1.42	0.42	4.78	0.57
Validation/test set	0.53	0.15	1.88	0.33

aOR adjusted Odds Ratios; *CI* Confidence Intervals; *NIHSS* National Institutes of Health Stroke Scale; *ICH* Intracerebral hemorrhage; *AHT* Arterial hypertension; *ICH* Intracranial hemorrhage; *mRS* modified Ranking Scale; *EVT* Endovascular thrombectomy; *IVT* Intravenous thrombolysis

Supplementary Table VII Adjusted Odds Ratios for death at 90 days (mRS 6) of multivariate logistic regression including predicted NIHSS >5

	OR	95% CI lower	95% CI upper	p
**Predicted NIHSS >5**	**1.92**	**0.50**	**7.38**	**0.34**
Age	1.07	1.00	1.14	0.03
Female sex	1.02	0.30	3.53	0.97
MedHist stroke	0.24	0.02	2.66	0.25
MedHist AHT	2.00	0.43	9.31	0.38
Diabetes	1.08	0.23	5.08	0.92
Smoking status	0.94	0.18	4.78	0.94
Hyperlipidemia	0.52	0.14	1.98	0.34
Atrial fibrillation	1.37	0.41	4.53	0.61
First Glucose (mmol/l)	1.07	0.86	1.32	0.56
Onset to imaging (min)	1.00	0.99	1.00	0.09
Prestroke mRS	1.54	0.90	2.64	0.12
Side of vessel occlusion	2.30	0.73	7.33	0.16
Affected vessel	0.97	0.52	1.83	0.94
EVT	4.60	0.45	46.72	0.20
IVT	1.33	0.40	4.46	0.64
Validation/test set	0.44	0.12	1.65	0.22

aOR adjusted Odds Ratios; *CI* Confidence Intervals; *NIHSS* National Institutes of Health Stroke Scale; *ICH* Intracerebral hemorrhage; *AHT* Arterial hypertension; *ICH* Intracranial hemorrhage; *mRS* modified Ranking Scale; *EVT* Endovascular thrombectomy; *IVT* Intravenous thrombolysis

Supplementary Methods I

In short, a dedicated stroke protocol was performed either on a 1.5T or 3T system including: A whole brain DWI (slice thickness 4-5 mm), an axial FLAIR (slice thickness 3-5 mm), TOF-MRA (slice thickness 0.5-0.6 mm), and an axial SWI (slice thickness 1.6-2 mm). After i.v. application of Gadobutrol (Gadovist; Bayer Healthcare, Berlin, Germany) in an antecubal vein with 5 ml/s injection rate a standard dynamic susceptibility contrast MR perfusion (slice thickness 5 mm) was acquired as well as a contrast-enhanced T1-weighted Sequence (slice thickness 4-5 mm). Finally, a contrast-enhanced MR angiography of the head and neck vessels was acquired after injection of a second bolus of Gadobutrol with a 3 ml/s injection rate.

Field strength	1.5T	3T
MRI scanner	Avanto, Aera	Skyra, Prisma, Verio, Vida
Sequences in stroke protocol	-ax DWI<br>-ax FLAIR<br>-ax SWI<br>-ax intracranial TOF<br>-ax DSC perfusino<br>-ax T1w post-contrast<br>-cor extracranial dynamic contrast-enhanced MRA	-ax DWI<br>-ax FLAIR<br>-ax SWI<br>-ax intracranial TOF<br>-ax DSC perfusino<br>-ax T1w post-contrast<br>-cor extracranial dynamic contrast-enhanced MRA
Scanner parameters	TR 49 ms, TE 40 ms, flip angle 15.0°, section thickness 1.6/1.8/2 mm, intersection gap 0 mm	TR 27 ms, TE 20 ms, flip angle 15.0°, section thickness 2 mm, intersection gap 0 mm

MRI Magnetic Resonance Imaging; *T* Tesla; *TR* Time to repetition; *TE* Time to echo.
Device manufacturer: Siemens Healthineers GmbH, Erlangen, Germany. All the scanners were upgraded 2016-2018 with slight modifications of scanning parameters.

Supplementary Methods II SUPER preprocessing pipeline (adapted from Zheng et al., 2026)

During the preprocessing stage, the raw sequence is registered to the first frame for motion correction. The skull is removed using the HD-BET for MRP using the first image of the sequence. To follow, the image intensity I of the MRP is converted to concentration time curve c(t) using the formula

$$c(t) = -\frac{k}{TE}\ln\left(\frac{I(t)}{I_0}\right)$$

where k is a correction factor, TE is the echo time, I(t) is the signal intensity at time t, and I_0 is the intensity of the first image of the sequence. A time-wise Gaussian filter is applied to c(t) at the pixel level that helps reduce noise without compromising the spatial resolution. A moving average with window size of three in time is applied subsequently to smooth c(t).

For MRP imaging, a background-removal step is performed by subtracting the average of the first three images from the entire sequence.

To capture the global advection process of the contrast agent, the preprocessed sequences are spatially averaged over the volume at each time point, yielding a single concentration time curve C(t). The time derivative of C(t) is then calculated to identify the last zero-crossing before the peak and the first zero-crossing after the peak, indicating the beginning and the end of the advection process. The volumes within this time interval are extracted from the sequence. A quadratic spline interpolation is applied at the pixel level, generating five additional volumes before and after the peak, respectively. The choice of five volumes is to balance between accuracy and computational efficiency. As a result, all sequences are regularized into a standardized series of eleven volumes that span the entire perfusion process.

To quantify perfusion delay, a peak-finding algorithm is applied to the regularized sequences. First, the integer index of the peak is identified. Then, a quadratic regression is performed using the two neighboring points to determine the precise peak position with sub-index resolution. The process is conducted at the pixel level, producing a volumetric map of peak times. This map, referred to as the surrogate TMAX map, quantifies the relative timing of peak concentration for each pixel. To follow, the surrogate map for CBV is calculated by integrating the regularized concentration curve C^* (t) at each pixel, while surrogate CBF by averaging the gradient of C^* (t) before the peak. Finally the surrogate MTT is calculated by CBV/CBF. These surrogate perfusion maps do not quantitatively match the perfusion maps acquired by deconvolution algorithms but nevertheless retain the same physical meaning of the perfusion process.

The four surrogate perfusion maps were taken as the input to train the nnU-Net for tissue at risk segmentation.

Both models employed the '3d_full_res' configuration from nnU-Net. GT labels for hypoperfused cases were manually labeled by board-certified neuroradiologists, and 10% of the data was reserved for testing. The remaining data was randomly split into 80% for training and 20% for validation. The loss function was the sum of Dice Score Coefficient (DSC) and Cross-Entropy loss. Optimization was performed using stochastic gradient descent with Nesterov momentum. The results were validated against Rapid and Cercare on external data. Full results can be found in our separate work: A fully automated framework (SUPER) with surrogate perfusion maps to assess hypoperfusion in ischemic stroke and SUPER: surrogate perfusion maps for infarct core assessment in ischemic stroke.
